# Supplementary material for: Novel Driver Strength Index highlights important cancer genes in TCGA PanCanAtlas patients
Source: PeerJ. 2022 Aug 11;10:e13860. doi: 10.7717/peerj.13860 (PMC9375969; doi:10.7717/peerj.13860)
Supplement: Supplemental Information 2 [file peerj-10-13860-s002.zip › COHORTS/genes plots/age/2021_8_16_14_9_distribution_cohorts_80-84.pdf]

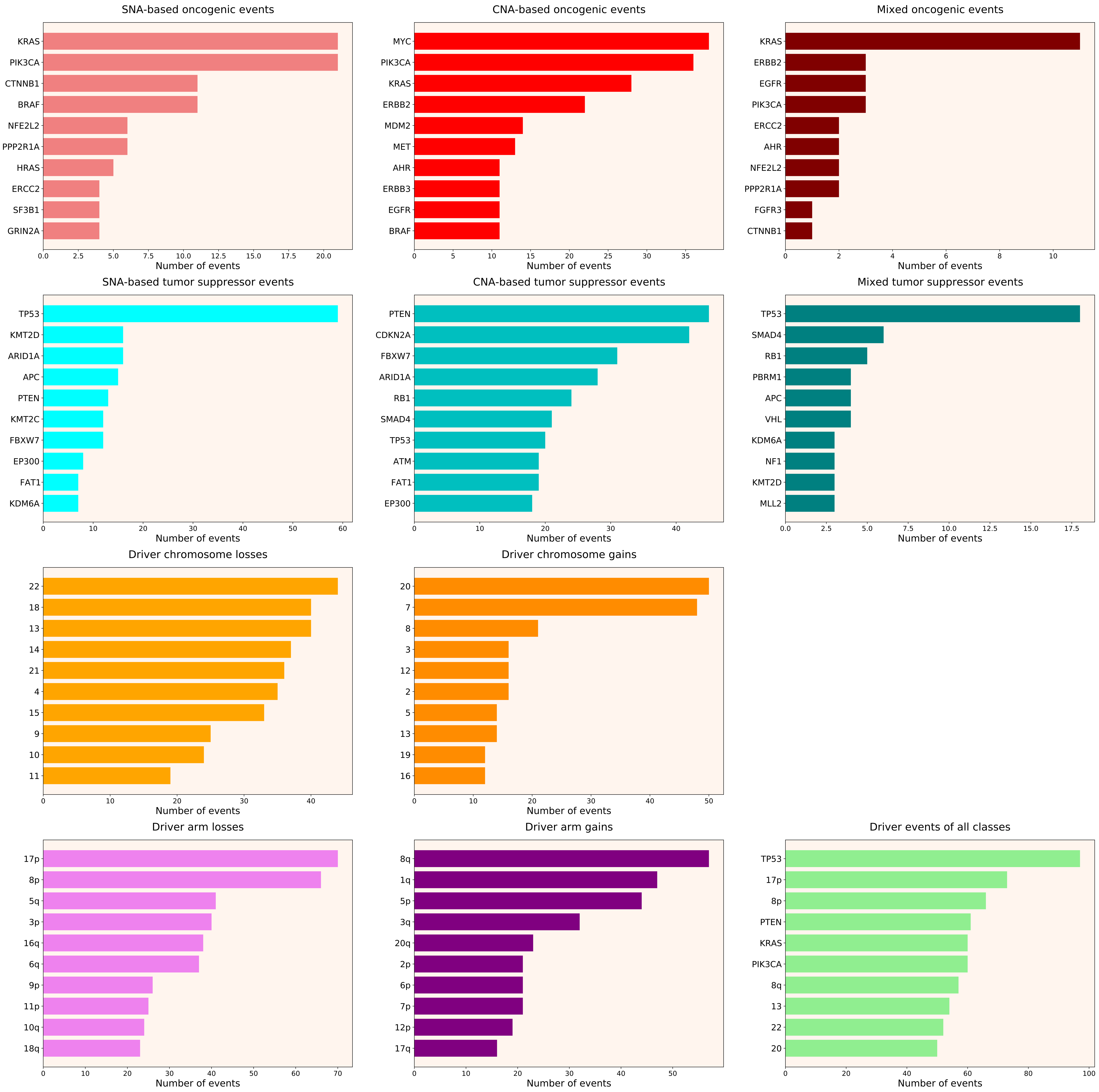
SNA-based oncogenic events
KRAS
PIK3CA
CTNNB1
BRAF
NFE2L2
PPP2R1A
HRAS
ERCC2
SF3B1
GRIN2A
0.0 2.5 5.0 7.5 10.0 12.5 15.0 17.5 20.0
Number of events
CNA-based oncogenic events
MYC
PIK3CA
KRAS
ERBB2
MDM2
MET
AHR
ERBB3
EGFR
BRAF
0 5 10 15 20 25 30 35
Number of events
Mixed oncogenic events
KRAS
ERBB2
EGFR
PIK3CA
ERCC2
AHR
NFE2L2
PPP2R1A
FGFR3
CTNNB1
0 2 4 6 8 10
Number of events
SNA-based tumor suppressor events
TP53
KMT2D
ARID1A
APC
PTEN
KMT2C
FBXW7
EP300
FAT1
KDM6A
0 10 20 30 40 50 60
Number of events
CNA-based tumor suppressor events
PTEN
CDKN2A
FBXW7
ARID1A
RB1
SMAD4
TP53
ATM
FAT1
EP300
0 10 20 30 40
Number of events
Mixed tumor suppressor events
TP53
SMAD4
RB1
PBRM1
APC
VHL
KDM6A
NF1
KMT2D
MLL2
0.0 2.5 5.0 7.5 10.0 12.5 15.0 17.5
Number of events
Driver chromosome losses
22
18
13
14
21
4
15
9
10
11
0 10 20 30 40
Number of events
Driver chromosome gains
20
7
8
3
12
2
5
13
19
16
0 10 20 30 40 50
Number of events
Driver arm losses
17p
8p
5q
3p
16q
6q
9p
11p
10q
18q
0 10 20 30 40 50 60 70
Number of events
Driver arm gains
8q
1q
5p
3q
20q
2p
6p
7p
12p
17q
0 10 20 30 40 50
Number of events
Driver events of all classes
TP53
17p
8p
PTEN
KRAS
PIK3CA
8q
13
22
20
0 20 40 60 80 100
Number of events
